# Plasma fibrinogen: a sensitive biomarker for the screening of periprosthetic joint infection in patients undergoing re-revision arthroplasty

**DOI:** 10.1186/s12891-022-05476-6

**Published:** 2022-06-01

**Authors:** Hong Xu, Li Liu, Jinwei Xie, Qiang Huang, Yahao Lai, Zongke Zhou

**Affiliations:** 1 Department of Orthopaedic Surgery, West China Hospital, Sichuan University, No.37, Guoxue Road, Wuhou District, Chengdu, 610041 Sichuan China; 2 Department of Orthopaedic Surgery, West China Hospital, Sichuan University/West China School of Nursing, Sichuan University, No.37, Guoxue Road, Wuhou district, Chengdu, 610041 Sichuan China

**Keywords:** Periprosthetic joint infection, Screening, Re-revision arthroplasty, C-reactive protein, Plasma fibrinogen, Neutrophil–lymphocyte ratio, Erythrocyte sedimentation rate

## Abstract

**Background:**

Although serum C-reactive protein (CRP), erythrocyte sedimentation rate (ESR), plasma fibrinogen and neutrophil–lymphocyte ratio (NLR) are promising biomarkers for screening PJI in patients undergoing revision arthroplasty, their efficacy with respect to re-revision arthroplasty remains unclear.

**Methods:**

We included patients who underwent re-revision arthroplasty at our hospital during 2008–2020, and stratified them into two groups whether they had been diagnosed with PJI (infected) or aseptic failure (non-infected) according to the 2013 International Consensus Meeting criteria. We evaluated the diagnostic performance of CRP, ESR, fibrinogen and NLR, both individually and in combinations, based on sensitivity, specificity, and area under the receiver operating characteristic curve.

**Results:**

Of the 63 included patients, 32 were diagnosed with PJI. The area under the ROC curve was 0.821 for CRP, 0.794 for ESR, 0.885 for fibrinogen and 0.702 for NLR. CRP gave a sensitivity of 87.5% and specificity of 74.2% with an optimal predictive cut-off of 8.50 mg/mL. ESR gave a sensitivity of 81.3% and specificity of 71.0% with an optimal predictive cut-off of 33 mm/h. Plasma fibrinogen gave a comparatively higher sensitivity of 93.8% and specificity of 77.4% with an optimal predictive cut-off of 3.55 g/L, while NLR gave a moderate sensitivity of 84.4% but low specificity of 54.8% with an optimal predictive cut-off of 2.30. The combination of fibrinogen and CRP gave a high AUC of 0.897, an acceptable sensitivity of 75% and a high specificity 93.5%.

**Conclusions:**

Plasma fibrinogen is a cost-effective, convenient biomarker that can be used to rule out PJI in patients scheduled for re-revision arthroplasty. In combination with CRP, it may be effective in diagnosing PJI in such patients.

## Introduction

Total hip and knee arthroplasty are increasingly used as effective procedures for the treatment of end-stage hip and knee diseases; it is estimated that approximately 4 million procedures will be performed in United States by 2030 [1]. However, periprosthetic joint infection (PJI) [2], periprosthetic fracture [3], aseptic loosening [4], and recurrent dislocation [5] are troublesome complications after total hip and knee arthroplasty. Although revision arthroplasty can reliably address these complications, its failure rate is high up to 22.8%, which is significantly higher than that of primary arthroplasty [6, 7].

PJI is the main cause of failure after revision arthroplasty, and it is strongly associated with extended treatments, increased hospitalization costs, as well as high morbidity and mortality rates [8–10]. A reliable diagnosis of PJI is extremely important for planning and implementing treatment regimens, preserving joint function, and managing patient expectations. It is especially important for patients who need to undergo re-revision arthroplasty since these patients often experience bone loss and scarring of skin, and their general health is poor [11, 12]. Therefore, it is critical to identify an effective blood biomarker that can be used to diagnose PJI in these patients reliably and in a relatively non-invasive manner.

Based on the recommendations of the 2013 International Consensus Meeting on PJI [13], serum C-reactive protein (CRP) and erythrocyte sedimentation rate (ESR) are used as biomarkers for the diagnosis of PJI in revision arthroplasty. However, the levels of these biomarkers may not rise following infection by weakly virulent pathogens such as *Propionibacterium acnes* [14]. Furthermore, the ability of these two biomarkers to screen for infection in re-revision arthroplasty remains unclear.

Plasma fibrinogen and neutrophil–lymphocyte ratio (NLR) have been associated with inflammation and infection [15, 16]. For example, NLR has been reported to accurately predict the occurrence and severity of community-acquired pneumonia [17]. Studies have reported that plasma fibrinogen is a promising biomarker for the diagnosis of PJI before revision arthroplasty [18–20], and that NLR is a reliable biomarker for diagnosis of early PJI after primary total knee and hip arthroplasty [21]. However, to our knowledge, the diagnostic performance of these biomarkers to identify PJI before re-revision arthroplasty has not been evaluated.

Therefore, in this single-center, retrospective cohort study, we analyzed data collected from patients who underwent re-revision arthroplasty at our hospital to evaluate the ability of plasma fibrinogen, NLR, CRP, and ESR – considered individually or in combinations – to identify PJI in patients who are scheduled for re-revision arthroplasty.

## Methods

### Study design

This single-center retrospective study was approved by the Institutional Review Board of West China Hospital, Sichuan University. The Institutional Review Board waived the requirement for written informed consent since the retrospective nature of this study posed no adverse effects to the health of patients, and patient data were anonymized during analysis.

### Patients

We recruited consecutive patients who underwent re-revision knee or hip arthroplasty for failure after one- or two-stage revision arthroplasty at our hospital between January 2008 and September 2020. We excluded the patients who were diagnosed with periprosthetic fracture or dislocation, and included those who underwent re-revision arthroplasty due to PJI or aseptic failure in the final analysis.

We used the diagnostic criteria for PJI recommended by the 2013 International Consensus Meeting on PJI [13] to stratify patients into two groups: those who had been diagnosed with PJI (infected group) and those who had been diagnosed with aseptic failure (non-infected group). In order to avoid missing infected patients, follow-up was conducted for the non-infected patients in the clinic or via telephone for ≥ 6 months.

### Laboratory evaluation

Based on routine preoperative testing, we collected data on serum CRP, ESR, plasma fibrinogen, and complete blood counts, including counts of neutrophils and lymphocytes, for all patients. In the case of patients with suspected PJI before re-revision arthroplasty, the joint under consideration was aspirated under sterile conditions on the day of admission or the following day. PJI was suspected based on medical history (especially the diagnostic workup before the first revision arthroplasty), levels of CRP and ESR, as well as clinical characteristics, such as symptoms and signs observed in X-ray images and during physical examinations as well as radiography.

Ultrasound-guided hip joint aspiration was performed by experienced technicians from the Doppler Ultrasound Department at our hospital, while knee aspiration was performed by surgeons in the aspiration room of the ward. The synovial fluid obtained in aspiration was then sent for laboratory evaluation. In addition to determining white blood cell counts, neutrophil differential counts, and polymorphonuclear neutrophil percentages, the laboratory performed aerobic and anaerobic cultures on blood agar. However, only cultures were performed if the volume of obtained synovial fluid was limited. Furthermore, synovial fluid was collected from all patients during the surgical procedure and cultured on blood agar. Cultures were routinely monitored for five days, however, in the case of suspected infection but no pathogen is identified, cultures were maintained for three weeks.

For each patient, biopsies were performed at two or more soft tissue locations around the implant, and these tissue samples were sent for histology analysis. The neutrophil counts of these samples were determined through careful observation under a microscope: if there were > 5 neutrophils per high-power field in a total of five high-power fields (× 400), the result was considered as positive.

### Outcomes and data extraction

The primary outcomes included levels of serum CRP, ESR, plasma fibrinogen, and NLR (ratio of the neutrophil to lymphocyte counts) before re-revision surgery. For all included patients, we extracted the following data from electronic medical records: (1) demographic data, such as age and sex; (2) the involved joint, primary diagnosis of the primary and first revision arthroplasties, use of antibiotics in the two weeks before current admission, as well as comorbidities such as hypertension, diabetes, chronic obstructive pulmonary disease, coronary heart disease, and inflammatory diseases; (3) results of laboratory and pathology tests; and (4) analysis of synovial fluid cultures. Additionally, the patients with inflammatory diseases such as rheumatoid arthritis and psoriasis were included in our analyses because of the confounding effects and the limited sample size [22].

### Sample size estimation

Minimum sample size was estimated using MedCalc 12.7 (MedCalc Software Ltd., Ostend, Belgium). For the biomarkers in the present study, previous work reported the following areas under the receiver operating characteristic curve (AUCs) for identifying PJI: CRP, 0.887; ESR, 0.842; plasma fibrinogen, 0.834 [18]; and NLR, 0.802 [21]. Therefore, we used the smallest AUC (0.802) to calculate the minimum sample size with a type I (significant) error of 0.05 and a type II (1-power) error of 0.1. Based on our estimation, the minimum sample size for each group was 17.

### Statistical analyses

All statistical analyses were performed using SPSS 24 (IBM, Armonk, NY, USA). Differences in normally distributed variables were compared using the Student’s t-test and expressed as mean and standard deviation (SD), while differences between variables that were skewed or showed unequal variance were compared using the Wilcoxon Mann–Whitney U test and expressed as median and interquartile range (IQR). Categorical variables were analyzed using Pearson’s chi-squared test and Fisher’s exact test, and expressed as frequency and percentages. A P value < 0.05 was considered to indicate statistical significance.

Receiver operating characteristic curves were used to evaluate the relationships between the true-positive rate (sensitivity) and the false-positive rate (1-specificity), as well as determine AUC values and their corresponding 95% confidence intervals (CIs). We also calculated the positive predictive value (PPV) and the negative predictive value (NPV). Optimal predictive cut-offs were derived for each of the four markers based on the Youden index. Additionally, we evaluated the ability of CRP and ESR to diagnose PJI based on the cut-offs recommended by the 2013 International Consensus Meeting on PJI: 10 mg/L for CRP and 30 mm/h for ESR [13].

## Results

**Fig. 1:**
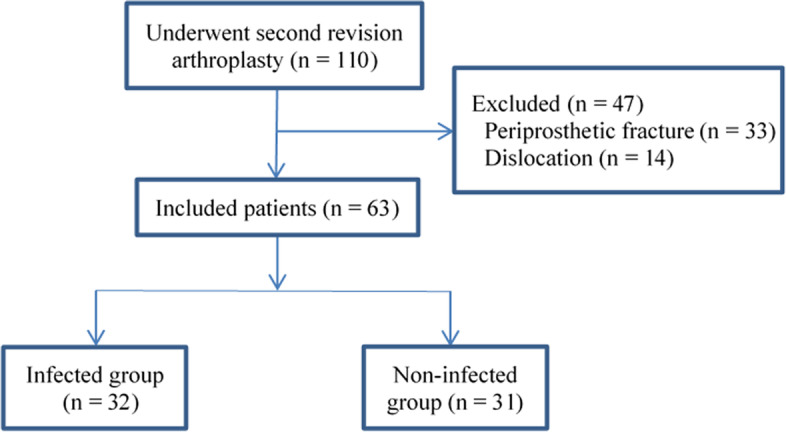
Flowchart of patient enrollment

A total of 90 patients who underwent re-revision arthroplasty at our hospital were recruited for this study. We excluded 13 patients with periprosthetic fracture and 14 with dislocation. The final analysis involved 63 patients, including 32 with PJI (infected group) and 31 with aseptic failure (non-infected) (Fig. 1).

The demographic characteristics and comorbidities of patients in both groups showed no significant differences (Table 1). Compared to the infected group, a significantly larger proportion of patients in the non-infected group underwent re-revision hip arthroplasty (96.7 vs 65.6%, *p* = 0.002; Table 1). The characteristics of patients in the infected group are listed in Table 2. More than 50% of patients in this group had been diagnosed with PJI before undergoing the first revision arthroplasty. There were 5 patients with inflammatory diseases, including 4 with rheumatoid arthritis and 1 with psoriasis. Altogether, 21 (65.6%) patients with a PJI were positive by culture tests. The most common pathogen causing infection among these patients was *Staphylococcus aureus*.

**Table 1:** Characteristics of the infected and non-infected groups

Variables	Infected group (*n* = 32)	Non-infected group (*n* = 31)	*P* value
Demographic characteristics			
Age, yr	64.14 ± 13.43	62.57 ± 11.54	0.620
Female	14 (43.8)	16 (51.6)	0.532
Comorbidities			
Hypertension	5 (15.6)	7 (22.6)	0.482
Diabetes	4 (12.5)	2 (6.5)	0.672
COPD	5 (15.6)	1 (3.2)	0.196
CHD	1 (3.1)	1 (3.2)	1.000
Inflammatory diseases	5 (15.6)	1 (3.2)	0.196
Involved joint (hip)	21 (65.6)	30 (96.7)	0.002^*^

Values are n (%) or mean ± SD, unless otherwise noted

*SLE* systemic lupus erythematosus, *COPD* chronic obstructive pulmonary disease, *CHD* coronary heart disease

^*^P < *0.05*

**Table 2:** The characteristics and the values of tested makers of the infected group

Patient	Involved joint	Diagnosis of the primary arthroplasty	Diagnosis of the first revision arthroplasty	Preoperative aspiration	Inflammatory diseases	Antibiotics use two weeks before current admission	Sinus tract	Pathogen (number of positive growths)	Histologic analysis	Serum CRP (mg/L)	ESR (mm/h)	Plasma FIB (g/L)	NLR	Synovial WBC (cells/μL)	Synovial PMN (%)
1	Hip	ONFH	PJI	Yes	-	Yes, orally	Yes	-	Positive	27.50	30	3.62	2.52	-	-
2	Hip	Femur neck fracture	PJI	Yes	-	Yes, intravenously	-	Escherichia coli (2)	Positive	80.00	25	4.54	8.00	-	-
3	Knee	Osteoarthritis	PJI	Yes	RA	No	-	-	Positive	23.10	15	3.57	2.25	4541	91
4	Knee	Osteoarthritis	PJI	Yes	RA	No	Yes	Staphylococcus aureus (3)	-	80.00	40	4.43	5.91	-	-
5	Hip	Femur neck fracture	PJI	Yes	-	No	-	-	Positive	16.40	46	5.5	1.35	> 20,000	92
6	Hip	ONFH	Aseptic loosening	Yes	-	Yes, intravenously	-	Enterobacter cloacae (6)	Positive	80.00	2	4.94	8.00	> 20,000	98
7	Hip	ONFH	Aseptic loosening	Yes	-	No	-	-	Positive	17.50	41	3.14	2.13	12,100	91
8	Hip	Femur neck fracture	Periprosthetic fracture	-	-	Yes, intravenously	Yes	-	-	7.11	45	3.61	5.70	-	-
9	Knee	Osteoarthritis	Aseptic loosening	Yes	-	Yes, intravenously	-	Proteus Peng (2)	Positive	1.09	10	3.74	5.79	-	-
10	Knee	Osteoarthritis	PJI	Yes	RA	Yes, intravenously	Yes	Staphylococcus aureus (2)	-	80.00	73	4.83	8.00	380	80
11	Hip	ONFH	PJI	Yes	-	No	Yes	Brucella citrate (1)	-	30.00	80	5.42	7.84	> 20,000	95
12	Hip	Femur neck fracture	Aseptic loosening	Yes	-	Unclear	-	Enterobacter urinalis (2)	Positive	26.50	67	4.45	2.53	1500	92
13	Hip	Femur neck fracture	Prosthesis fracture	Yes	Psoriasis	Unclear	Yes	Staphylococcus aureus (1)	Positive	58.80	120	4.23	3.65	-	-
14	Knee	Osteoarthritis	PJI	Yes	-	No	-	Staphylococcus aureus (2)	-	24.30	78	5.47	5.97	-	-
15	Knee	Osteoarthritis	PJI	Yes	-	No	-	Enterococcus faecalis (3)	-	62.40	79	4.72	3.25	78	48
16	Hip	Femur neck fracture	Periprosthetic fracture	Yes	-	Yes, intravenously	-	-	Positive	26.70	60	3.7	2.43	15,540	90
17	Knee	Osteoarthritis	Periprosthetic fracture	Yes	-	No	-	Enterobacter cloacae (2)	-	87.70	58	5.71	8.00	> 20,000	93
18	Hip	ONFH	PJI	Yes	-	Yes, intravenously	-	Enterococcus faecium (4)	Positive	61.30	80	5.5	3.98	-	-
19	Knee	Osteoarthritis	PJI	Yes	-	Yes, intravenously	Yes	Staphylococcus epidermidis (1)	-	75.00	70	3.98	1.89	> 20,000	90
20	Hip	ONFH	PJI	Yes	-	No	-	Escherichia coli (2)	-	21.20	68	4.62	2.08	-	-
21	Knee	Osteoarthritis	PJI	Yes	-	Yes, intravenously	-	Enterobacter cloacae (2)	Positive	48.80	85	4.77	2.72	> 20,000	95
22	Knee	Osteoarthritis	PJI	Yes	-	No	-	Enterococcus faecalis (3)	-	16.40	80	3.98	2.89	8770	90
23	Hip	Femur neck fracture	PJI	Yes	-	Yes, intravenously	-	Pseudomonas aeruginosa (2)	-	4.82	34	4.83	2.15	6671	98
24	Hip	Intertrochanteric fracture	PJI	Yes	-	Yes, intravenously	-	Pseudomonas aeruginosa (3)	Positive	19.30	70	4.96	3.12	-	-
25	Hip	Femur neck fracture	PJI	Yes	-	Yes, orally	-	Streptococcus parisiae (4)	Positive	80.00	80	5.5	7.78	3560	98
26	Hip	ONFH	Aseptic loosening	Yes	-	Unclear	Yes	-	Positive	9.06	51	3.67	2.65	-	-
27	Knee	RA	Periprosthetic fracture	Yes	RA	Yes, intravenously	Yes	-	-	18.00	78	4.81	3.51	7400	95
28	Hip	ONFH	Aseptic loosening	Yes	-	Yes, intravenously	-	-	Positive	10.50	52	3.93	2.75	12,750	91
29	Hip	ONFH	Aseptic loosening	Yes	-	No	Yes	-	-	5.20	36	2.57	3.20	-	-
30	Hip	ONFH	PJI	Yes	-	No	-	-	Positive	17.20	48	3.68	3.67	250	92
31	Hip	ONFH	Aseptic loosening	Yes	-	No	-	Staphylococcus epidermidis (2)	Positive	19.00	60	4.59	2.45	> 20,000	87
32	Hip	ONFH	Aseptic loosening	Yes	-	No	-	Staphylococcus epidermidis (4)	Positive	52.70	29	4.19	2.48	1200	95

*ONFH* osteonecrosis of the femoral head, *RA* rheumatoid arthritis, *PJI* periprosthetic joint infection, *CRP* C-reactive protein, *ESR* erythrocyte sedimentation rate, *FIB* fibrinogen, *NLR* neutrophil–lymphocyte ratio, *WBC*: white blood cell, *PMN* polymorphonuclear neutrophil

When we compared levels of the four biomarkers between the two groups, we found that patients in the infected group had significantly higher levels of CRP [25.40 (16.60–62.13) vs 4.08 (2.48–13.80), *P* < 0.001], ESR [59 (37–78) vs 19 (13–45), *P* < 0.001], plasma fibrinogen [4.41 ± 0.77 vs 3.19 ± 0.7, *P* < 0.001], and NLR [3.16 (2.46–5.88) vs 2.18 (1.56–3.44), *P* = 0.006] than patients in the non-infected group (Table 3).

**Table 3:** Tested markers in the infected and non-infected groups

Potential markers	Infected group (*n* = 32)	Non-infected group (*n* = 31)	*P* value
CRP (mg/L)	25.40 (16.60–62.13)	4.08 (2.48–13.80)	< 0.001^*^
ESR (mm/h)	59 (37–78)	19 (13–45)	< 0.001^*^
FIB (g/L)	4.41 ± 0.77	3.19 ± 0.70	< 0.001^*^
NLR	3.16 (2.46–5.88)	2.18 (1.56–3.44)	0.006^*^

*CRP* C-reactive protein, *ESR* erythrocyte sedimentation rate, *FIB* fibrinogen, *NLR* neutrophil–lymphocyte ratio

^*^Data were presented as median (interquartile range) or mean ± SD; ^*^P < *0.05*

**Fig. 2:**
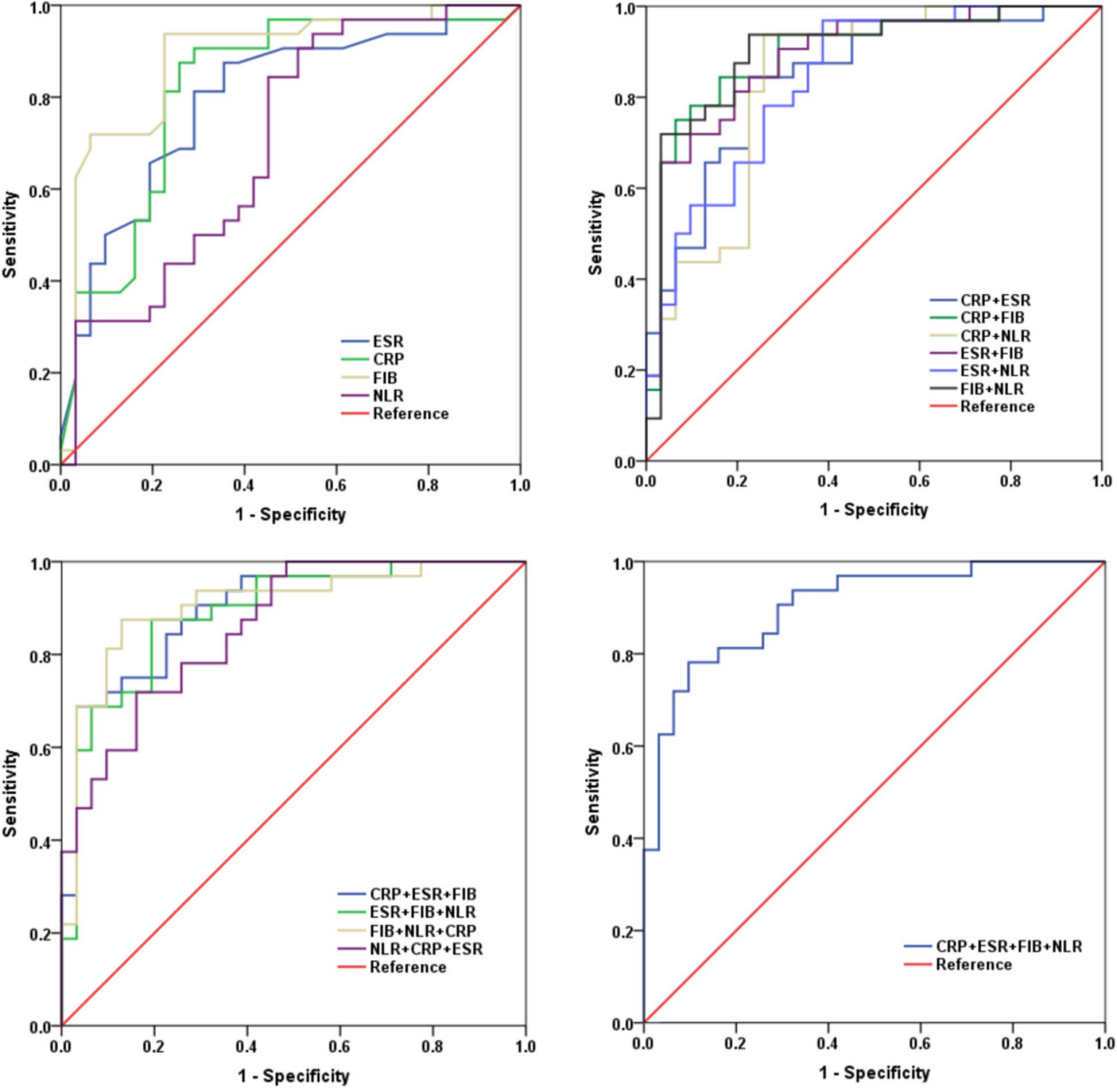
Receiver operating characteristic curves. **a** C-reactive protein (CRP), erythrocyte sedimentation rate (ESR), fibrinogen (FIB), neutrophil–lymphocyte ratio (NLR) on their own. **b** Combinations of two markers. **c** Combinations of three markers. **d** Combination of four markers

First, we evaluated the diagnostic ability of each of these biomarkers to identify PJI in patients who underwent re-revision arthroplasty. We found that plasma fibrinogen had the highest AUC (0.885, 95% CI 0.797–0.973), followed by CRP (0.821, 95% CI 0.712–0.930), ESR (0.794, 95% CI 0.690–0.907), and NLR (0.702, 95% CI 0.571–0.832) (Fig. 2a). CRP gave a sensitivity of 81.3% and specificity of 74.2% at the recommended cut-off of 10 mg/L [13], but it showed higher sensitivity (87.5%) and the same specificity (74.2%) at the optimal predictive cut-off of 8.50 mg/L, derived based on the Youden index. In contrast, ESR gave a sensitivity of 81.3% and specificity of 64.5% at the recommended cut-off of 30 mm/h [13], but it showed a higher specificity (71%) and the same sensitivity (81.3%) at the optimal predictive cut-off of 33 mm/h. The optimal cut-off for plasma fibrinogen was 3.55 g/L, which gave a high sensitivity (93.8%) and an acceptable specificity (77.4%). The optimal cut-off for NLR was 2.3, which gave a sensitivity of 84.4% and low specificity of 54.8% (Table 4). The highest NPV (92.4%) and acceptable PPV (81.1%) of fibrinogen confirmed its ability to screen for PJI before re-revision arthroplasty.

**Table 4:** Diagnostic performance of the tested markers individually

Potential markers	AUC (95% CI)	Youden index	Predictive cutoff	Sensitivity	Specificity	PPV	NPV
CRP (mg/L)	0.821(0.712–0.930)	0.555	10.00^a^	81.3%	74.2%	76.5%	79.4%
		0.617	8.50^b^	87.5%	74.2%	77.8%	85.2%
ESR (mm/h)	0.794(0.690–0.907)	0.458	30^a^	81.3%	64.5%	70.3%	77.0%
		0.523	33^b^	81.3%	71.0%	74.3%	78.6%
FIB (g/L)	0.885(0.797–0.973)	0.712	3.55^b^	93.8%	77.4%	81.1%	92.4%
NLR	0.702(0.571–0.832)	0.392	2.30^b^	84.4%	54.8%	66.0%	77.7%

*AUC* area under the receiver operating characteristic curve, *95% CI* 95% confidence interval, *CRP* C-reactive protein, *ESR* erythrocyte sedimentation rate, *FIB* fibrinogen, *NLR* neutrophil–lymphocyte ratio, *PPV* positive predictive value, *NPV* negative predictive value

^a^Predictive cutoffs determined based on the Musculoskeletal Infection Society criteria

^b^Predictive cutoffs determined based on the Youden index

Next, we evaluated the diagnostic ability of different combinations of the four biomarkers to identify PJI in our patients. Among the two biomarker combinations, we found that a combination of plasma fibrinogen and CRP was associated with a high AUC (0.897, 95% CI 0.816–0.978), an acceptable sensitivity (75%), and the highest specificity (93.5%); the highest PPV (92.3%) and acceptable NPV (78.4%) associated with this combination indicate its effectiveness in diagnosing PJI before re-revision arthroplasty. The combination of all four markers was associated with a high AUC (0.903, 95% CI 0.830–0.977), an acceptable sensitivity (78.1%), a high specificity (90.3%), a high PPV (89.3%) and acceptable NPV (80%) (Table 5, Fig. 2b-2d). In addition, the combinations of three or four biomarkers were not better at diagnosing PJI than fibrinogen, either on its own or combined with CRP. The combination of these two biomarkers appears more suitable for clinical use.

**Table 5:** Diagnostic performance of the tested markers in combination

Combinations	AUC (95% CI)	Youden index	Sensitivity	Specificity	PPV	NPV
Combination of two markers						
CRP + ESR	0.847(0.751–0.943)	0.618	84.4%	77.4%	79.5%	83.2%
CRP + FIB	0.897(0.816–0.978)	0.685	75.0%	93.5%	92.3%	78.4%
CRP + NLR	0.840(0.739–0.941)	0.680	93.8%	74.2%	79.0%	92.1%
ESR + FIB	0.890(0.809–0.971)	0.619	81.3%	80.6%	81.2%	80.7%
ESR + NLR	0.839(0.742–0.936)	0.582	90.6%	61.3%	70.7%	86.3%
FIB + NLR	0.900(0.818–0.982)	0.712	93.8%	77.4%	81.0%	92.1%
Combination of three markers						
CRP + ESR + FIB	0.896(0.819–0.973)	0.656	68.8%	96.9%	95.8%	75.1%
ESR + FIB + NLR	0.888(0.806–0.970)	0.681	87.5%	80.6%	82.3%	86.2%
FIB + NLR + CRP	0.905(0.827–0.984)	0.746	87.5%	87.1%	87.5%	87.1%
NLR + CRP + ESR	0.860(0.773–0.947)	0.558	71.9%	83.9%	82.2%	74.3%
Combination of four markers						
CRP + ESR + FIB + NLR	0.903(0.830–0.977)	0.684	78.1%	90.3%	89.3%	80.0%

*AUC* area under the curve, *95% CI* 95% confidence interval, *CRP* C-reactive protein, *ESR* erythrocyte sedimentation rate, *FIB* fibrinogen, *NLR* neutrophil–lymphocyte ratio, *PPV* positive predictive value, *NPV* negative predictive value

## Discussion

In this study, we assessed the diagnostic performance of four biomarkers – CRP, ESR, plasma fibrinogen, and NLR – to identify PJI in patients scheduled for re-revision arthroplasty. Our results suggest that plasma fibrinogen is a useful biomarker for ruling out PJI in patients scheduled for this procedure, and it may be effective, when combined with CRP, for diagnosing PJI in such patients. As far as we know, this is the first study to evaluate the diagnostic ability of plasma fibrinogen and NLR to identify PJI in patients who have undergone re-revision hip and knee arthroplasty.

Compared to primary revision arthroplasty, patients undergoing the re-revision procedure face significant challenges, including increased pain and financial stress. Surgeons also face a more complex operation due to bone loss, scarring of skin, and high tension in soft tissue. Failure after re-revision arthroplasty can cause distress among patients, and PJI is one of the main reasons for such failure [8, 12]. Therefore, highly sensitive biomarkers are needed to rule out infection before re-revision arthroplasty and reduce its incidence afterwards. Our study showed that plasma fibrinogen can be used as a sensitive biomarker to rule out PJI before re-revision (sensitivity 93.8%, specificity 77.4%), and that the specificity can increase to 93.5% if this biomarker is combined with CRP.

Plasma fibrinogen is familiar to surgeons because it is one the basic parameters of blood clotting functions that is routinely monitored before surgery. Fibrinogen is a large hexameric homodimer (340 kDa) that plays an important role in hemostasis and homeostasis [23]. It is synthesized and secreted by the liver, and this process is regulated at the transcriptional and translational levels [24, 25]. Since it is an acute phase protein, inflammation and infection can effectively promote its synthesis and secretion. Its concentration in the plasma can exceed 7 g/L during acute inflammation [15, 24]. These features indicate its potential to act as a biomarker for the screening of infection.

Although several studies have demonstrated the diagnostic value of plasma fibrinogen to identify PJI before revision arthroplasty [19, 26], as well as to identify infection in patients who experience non-union after open reduction and internal fixation [27], its efficacy with respect to re-revision arthroplasty has rarely been discussed. Our study revealed that, at an optimal cut-off of 3.55 g/L, plasma fibrinogen can be used as a sensitive biomarker to rule out PJI in patients undergoing re-revision arthroplasty (sensitivity 93.8%). This optimal cut-off is lower than the 4.01 g/L reported in a study to diagnose PJI before revision arthroplasty [19]. This difference may reflect that the present study examined *re*-revision arthroplasty and that the pathogens involved may differ in virulence. It may also reflect differences in laboratory procedures. Considering that this biomarker may be used as a first-line screening tool, a lower cut-off could reduce the rate of misdiagnosis in patients with PJI.

NLR has been associated with systemic inflammation and infection. One study reported that preoperative NLR ≥ 2.3 can be used to predict major surgical complications after colorectal resection [28]. In patients with odontogenic infection, the level of NLR was associated with the dose of antibiotics and length of hospital stay [29]. Furthermore, NLR can be used for the diagnosis of early PJI after total knee arthroplasty, since it normalizes faster than CRP [21, 30]. The present study showed that NLR, on its own, has limited diagnostic ability to screen for PJI in patients undergoing re-revision arthroplasty. However, the combination of NLR and CRP may be effective for such screening.

Besides clinical manifestations, the analysis of synovial fluid [31] and blood biomarkers [32] are the main tools for diagnosing PJI. Although synovial fluid cultures can be used to identify the pathogens that caused PJI, the procedure is complicated, especially when it involves the hip joint, and there is a risk of introducing bacteria into the joint and causing secondary infection [33]. In contrast, blood biomarkers have several advantages such as convenience, speed, and cost-effectiveness, and they therefore play a crucial role in screening for PJI, especially among outpatients. Our findings show that plasma fibrinogen can be used as a highly sensitive screening biomarker before re-revision arthroplasty. Plasma fibrinogen levels can be measured in most hospitals, and levels are tested routinely before surgery [19]. Thus, we propose plasma fibrinogen as a cost-effective, convenient biomarker to screen for PJI among patients undergoing re-revision arthroplasty.

These findings must be considered in the light of certain limitations. First, this is a single-center retrospective study, which means that our results may not be generalizable to other cohorts. Second, due to our small sample, we could not evaluate the effect of antibiotic use, comorbidities such as inflammatory diseases, or coagulation and blood disorders on the ability of these four biomarkers to identify PJI in patients undergoing re-revision arthroplasty. Further multi-center research must be conducted to validate and extend our findings.

## Conclusions

Our study showed that plasma fibrinogen is a cost-effective, convenient biomarker that can be used to rule out PJI in patients undergoing re-revision arthroplasty. This biomarker, when combined with CRP, may be specific enough to diagnose PJI in such patients.

## Data Availability

Please contact the first or correspondence author (Hong Xu or Zongke Zhou) for data requests.
